# Cardiac disease in pregnancy

**Published:** 2008-06

**Authors:** T Nqayana, J Moodley, DP Naidoo

**Affiliations:** Department of Obstetrics and Gynaecology, Nelson R Mandela School of Medicine, University of KwaZulu-Natal, Durban; Women’s Health and HIV Research Group, Nelson R Mandela School of Medicine, University of KwaZulu-Natal, Durban; Sub-department of Cardiology. Nelson R Mandela School of Medicine, University of KwaZulu-Natal, Durban

## Abstract

**Summary:**

This study was a retrospective review of patient charts of a relatively large number of patients with cardiac disease in pregnancy in a developing country. Ninety-five patients were evaluated; the majority (*n* = 36) were in the age group 21−25 years. Rheumatic heart disease was the commonest aetiology; eight women required balloon mitral valvuloplasty and one had a valve replacement at 32 weeks’ gestation. There were no maternal deaths but morbidity was high; 13 patients were admitted in cardiac failure, nine had atrial fibrillation and three required intensive-care management. There were 86 live births of the 97 deliveries.

## Summary

Cardiac disease in pregnancy is still a major problem worldwide, particularly in poor countries. Although the reported incidence varies between 0.1 and 4%,^1^-^3^ cardiac disease remains a significant cause of maternal death worldwide.^4^

Rheumatic heart disease (RHD) is the major cause of cardiac disease in poor countries, but is uncommon in affluent societies, resulting in few reports on large series of cases of cardiac disease in pregnancy from well-resourced countries. A previous study from Durban, South Africa, reviewed 128 cases of isolated mitral stenosis seen over a four-year period and highlighted the hazards of unrecognised valvular stenosis in pregnancy.^5^ However, most patients with cardiac disease in pregnancy, in our clinical experience, present with mixed mitral valve disease. Furthermore, pathological involvement of other cardiac valves in isolation or in combination is also associated with significant morbidity.

Recent changes in health service facilities in the Durban area have resulted in the majority of patients with cardiac disease being managed in one tertiary hospital. This has provided an opportunity to report on our clinical experiences of cardiac disease in pregnancy. Maternal and foetal outcomes of patients admitted with cardiac disease over a one-year period at Inkosi Albert Luthuli Central tertiary hospital, (IALCH) Durban, South Africa were evaluated.

## Methods

A retrospective analysis of all patients admitted with cardiac disease over a one-year period was performed. All patient data (in-patient and out-patient information) at IALCH is recorded onto a clinical software package (Medicom, Medicom Solutions, India). Information retrieved from this database included demographic details, clinical management, surgery performed pre-pregnancy and during pregnancy, and data on maternal and foetal outcomes. The data were collected in a structured format.

The usual policy at IALCH is that cardiac patients are admitted on the first visit for a complete clinical assessment. A detailed history is obtained and the patient’s disability graded according to the New York Heart Association classification.^6^ Blood investigations included a full blood count, serum renal function tests, voluntary testing for a rapid serum human immunodeficiency virus (HIV) and specific tests included ECG, chest X-ray and echocardiogram. A cardiologist, together with an obstetrician interested in medical complications of pregnancy assessed all patients and reviewed treatment regimens.

Patients with prosthetic metallic valves received heparin in the first trimester and this was converted to warfarin at 13 weeks gestational age and then reconverted to heparin at 36 weeks gestational age. Following initial assessment, patients who were in NYHA functional class I and II were managed as outpatients and followed up weekly. Those who were in NYHA class III and IV were hospitalised mainly due to poor social circumstances and the fact that most were referred from distant areas of the province.

If antenatal care was uneventful, decisions on mode of delivery and review by anaesthesiologists was taken at approximately 34 weeks’ gestation. Vaginal delivery with epidural analgesia for pain relief was the principle guiding the decision on mode of delivery. Caesarean section (C/S) was performed if vaginal delivery was contraindicated for obstetric reasons. All primigravidae had computerised tomographic pelvimetry to assess cephalopelvic disproportion, as trial of labour was not allowed in cardiac patients with an inadequate pelvis.

Maternal outcome was analysed using the following criteria: deterioration in NYHA functional class, cardiac complications, non-cardiac complications, cardiac interventions during pregnancy and mode of delivery. Neonatal outcome was analysed using the following criteria: time of delivery, birth weight, stillbirth, congenital abnormalities and congenital pneumonia.

## Statistics

Descriptive statistics were utilised and all results are presented as frequencies, means and percentages. Correlation coefficients and multiple regressions for echocardiographic data analysis were applied.

## Results

Ninety-five patients with cardiac disease were seen during the study period of a year. The demographic data are shown in Table 1. Cardiac disease was detected for the first time during preg- nancy in 23 cases, 18 of which had RHD. There was one case of twin pregnancy; 72 patients were under 30 years old and most were multiparous (*n* = 53). The majority (*n* = 58) presented for antenatal care in the third trimester and six delivered at ≤ 32 weeks’ gestation.

**Table 1.** Demographic Data Of All Patients

*Characteristics*	*No of patients (n = 95)*
Age (years)	
15−20	12
21−25	36
26−30	24
31−40	18
> 40	5
Parity	
Primigravida	38
P1−3	53
≥ P4	4
Previous miscarriage	11
Gestational age at admission	
1st trimester	6
2nd trimester	31
3rd trimester	58
Gestational age at delivery (weeks)	
< 28	2
28−32	7
34−36	26
Term	60
HIV positive	31
CD_4_ count < 200 cells/ml	3
NYHA class	
I	38
II	33
III	16
IV	8

NYHA = New York Heart Association.

The anatomical and pathological valve lesion in all 95 patients is shown in Table 2. Rheumatic heart disease was the underlying pathological condition in 81% (*n* = 77) of cases. There were 71 in functional class (NYHA) I−II and 24 in class III−IV. Congenital heart disease accounted for 9.5% (*n* = 9) of cases. In addition to cardiac disease, 19 patients had associated medical conditions: hypertension (*n* = 7) was the most prevalent, followed by asthma (*n* = 3); diabetes (*n* = 1) and epilepsy (*n* = 1); the remaining seven had chest infections and urinary tract infections.

**Table 2.** Clinical Spectrum Of Cardiac Disease

*Cardiac lesion*	*No of patients (n = 95)*
Rheumatic heart disease	77
Mitral stenosis	22
Mixed mitral valve	17
Mitral regurgitation	11
Aortic regurgitation	1
Aortic stenosis	1
Multiple valve lesion	5
Metallic valve replacement	20
Congenital heart disease	9
Atrial septal defect	2
Ventricular septal defect	5
Patent ductus arteriosus	1
Tetralogy of Fallot	1
Myocardial/other	9

Echocardiograms were performed in all patients. The ejection fraction was normal in all except three with cardiomyopathy. The mitral valve area ranged from 0.3−2.8 cm^2^. The severity of mitral stenosis was graded according to mitral valve area, as described by Desai *et al*. 2000.^5^ Echo-Doppler studies identified seven patients as having moderately severe to critical mitral stenosis (mitral valve area 0.8−1.2 cm^2^), changing the clinician’s diagnosis of mixed mitral valve disease.

Although there were no maternal deaths during the study period, a significant number of cardiac and non-cardiac complications were encountered in 32 patients (Table 3). Cardiac complications occurred in 30 patients and were mainly related to rhythm disturbances and heart failure. Bleeding from over-anticoagulation accounted for the majority of the non-cardiac complications. Outcomes are discussed under the following categories: rheumatic heart disease, congenital heart disease and myocardial disease.

**Table 3.** Complications Associated With Cardiac Disease In Pregnancy

*Complication*	*No of patients*
Cardiac	
Atrial fibrillation	9
Heart failure	13
Intensive care admission	3
Infective endocarditis	2
Pulmonary embolism	1
Supraventricular tachycardia	3
Hypercoagulation	3
Severe hypertension	1
Wound haematoma	2
Cerebral infarct	1
Non-cardiac	
Puerperal sepsis	4
Broad ligament haematoma	1
Postpartum haemorrhage	5
Anaemia (Hb < 9 g/dl)	16
Thrombocytopenia (Plt < 150 3 10^9^/l)	15

## Rheumatic heart disease group

*Rheumatic valve disease:* Of the 77 patients in this group, 26 had a corrective surgical measure performed prior to pregnancy and 18 had newly diagnosed cardiac disease. The remainder were known to have valvular disease.

*Metallic valve replacement:* Twenty patients had metallic valve replacement prior to pregnancy (Table 2). All but six had their first visit in the second/third trimester and therefore did not receive heparin in the first trimester. One had defaulted on follow up with her physician and was not on any anticoagulants. Two were symptomatic (NYHA III−IV); one presented in heart failure from a clotted valve and the other had a leaking prosthesis. The patient who presented in cardiac failure at 32 weeks, secondary to a thrombosed prosthetic valve, required an emergency valve replacement. During the procedure, she went into labour and delivered a stillborn foetus.

Maternal complications were noted in 10 patients: two had atrial fibrillation and wound haematoma, two had atrial fibrillation antenatally and postpartum haemorrhage later, two had congestive cardiac failure (described below), two had postpartum haemorrhage, and in another two, there were problems related to poorly controlled anticoagulation.

Five patients delivered prematurely at gestations of ≤ 36 weeks: three had an elective C/S, one had an emergency C/S, and one was delivered by forceps. Five patients had adverse foetal outcomes: one had a miscarriage; one had a neonatal death associated with a congenital anomaly (gastroschisis); three had stillbirths (two of whom had gross congenital anomalies). Another three babies had low birth weights (< 2 kg).

*Stenotic valve lesions:* There were 22 patients with mitral stenosis and one with aortic stenosis; 10 patients with mitral stenosis were diagnosed for the first time during pregnancy and 11 were in NYHA class III−IV. Four patients had undergone balloon mitral valvuloplasty (BMV) prior to pregnancy. Eight patients required BMV during pregnancy, in three of these it was performed for restenosis following BMV.

Five patients presented in cardiac failure and another five had atrial fibrillation. One of the patients in congestive cardiac failure was at 31 weeks of gestation. She had a tight pliable mitral stenosis (valve area 0.8 cm2) with pulmonary hypertension. She had a scoliosis and was in respiratory acidosis, necessitating ventilation. She also had pre-eclampsia with foetal compromise and thrombocytopaenia. She had an emergency C/S and delivered a 1.54-kg infant. There were five others with low birth weights (2−2.4 kg). The patient who had severe aortic stenosis and mitral stenosis delivered a low-birth weight infant at term.

Of the 22 patients with mitral stenosis, 14 delivered by C/S, (five had emergency C/S) and eight had vaginal deliveries. One of the patients who had balloon mitral valvuloplasty in pregnancy had spontaneous rupture of membranes following the procedure and delivered a live infant. One patient had an intrauterine death following an amniocentesis done on the basis of advanced maternal age.

Mixed mitral valve disease: Of the 17 (22%) patients with mixed mitral valve disease, 13 were known to have valvular disease and four were newly diagnosed during the pregnancy. Two patients had undergone balloon mitral valvuloplasty prior to pregnancy for tight mitral stenosis. Four patients were in NYHA class III−IV. Three of the four newly diagnosed patients developed complications: congestive cardiac failure (one), severe hypertension (one) and puerperal sepsis (one). All in this group responded to medical treatment and surgical intervention was not required.

A further four patients developed complications as follows: congestive cardiac failure following on infective endocarditis (one), atrial fibrillation (one), wound haematoma (one) and cerebrovascular accident (one). The patient who had a cerebrovascular accident was a primigravida who had a mixed lesion with predominant stenosis (mitral valve area: 1.2 cm^2^). She had an elective C/S at 37 weeks for oligohydramnios with a borderline pelvis and delivered a live 2.5-kg infant. On day 28, she presented with aphasia and a right hemiplegia and CT scan revealed an infarct in the left middle cerebral artery territory; an echocardiogram showed no thrombi.

Ten patients delivered at term and the remainder delivered at 29−36 weeks’ gestation. Twelve were delivered by elective C/S (three had emergency C/S) and there were five vaginal deliveries. There was one stillbirth in this group; the patient presented in advanced pre-term labour at 29 weeks with an intrauterine death.

Regurgitant valvular lesions: This group of 17 patients constituted those who had pure mitral regurgitation (*n* = 11), mitral regurgitation with aortic regurgitation (*n* = 4) and the other lesions were aortic regurgitation (*n* = 1) and mitral regurgitation with tricuspid regurgitation (*n* = 1). Two were NYHA class III−IV and responded to medical therapy. One had congestive cardiac failure and a second had supraventricular tachycardia with infective endocarditis in the antenatal period. Of the 17 in this group, three patients were newly diagnosed.

Eight patients were delivered electively by C/S, one by emergency C/S, and 10 were delivered vaginally. One patient who had supraventricular tachycardia was noted to have intrauterine growth restriction at 36 weeks’ gestation and delivered a live 2.02-kg infant with a good Apgar score by elective C/S.

## Congenital heart disease group

Eight of the nine patients in this group were evaluated during the antenatal period and were all in NYHA class I−II. One had an uncorrected ventriculoseptal defect diagnosed for first time postpartum when she presented in cardiac failure. None had pulmonary hypertension. Six had vaginal delivery; two had an elective C/S and one had an emergency C/S. One patient had postpartum haemorrhage.

Two babies had complications, namely, birth asphyxia, which responded to resuscitation, and an early neonatal death, respectively. The neonatal death occurred in a mother who had a ventricular septal defect; she went into spontaneous labour at 39 weeks’ gestation. The progression of labour was uneventful but she delivered an infant with an Apgar score of 3/10. A paediatrician had difficulty intubating the baby due to upper airway obstruction and the baby demised two hours later.

## Myocardial group

This group of nine patients comprised cardiomyopathy (*n* = 3) (presented in cardiac failure), hypertensive heart disease (*n* = 4), Graves disease (*n* = 1) and pulmonary embolism (*n* = 1). One required intensive-care monitoring post-delivery. She had an ejection fraction of 30%, and required inotropic support with dobutamine. She had a persistent tachycardia that subsided when carvedilol was added to her management. An elective C/S was performed and she was managed postoperatively in the ICU with an uneventful recovery. Five were delivered by C/S, three had normal vaginal deliveries and one with cardiomyopathy had a miscarriage. One hypertensive patient delivered a pre-term baby.

## Pregnancy outcome

Since assisted vaginal delivery was construed as the most ideal outcome for both mother and baby, pregnancy outcome was arbitrarily classified in descriptive terms, namely, good, satisfactory and poor; taking into account the mode of delivery and the foetal outcome as follows: good outcome − vaginal delivery, no maternal/foetal adverse effects; satisfactory outcome − C/S performed with no maternal/foetal adverse effects; and poor outcome − maternal and/or foetal adverse effect, irrespective of mode of delivery. The overall pregnancy outcome was satisfactory (Table 4).

**Table 4.** Pregnancy Outcome VS Cardiac Lesion

	*Good*	*Satisfactory*	*Poor*
Metallic valve	1	9	10
Mixed mitral valve	4	11	2
Mitral stenosis	6	12	14
Mitral regurgitation	7	4	0
Multi-valve disease	3	3	1
Congenital heart disease	4	2	3
Myocardial disease	4	3	2

In the native valve and prosthetic valve groups, only 21/77 (27%) patients had a good outcome, illustrating the significant morbidity associated with these valve lesions. Patients in the mitral regurgitation group had a much better outcome than the other groups. In the congenital and myocardial group, the number of patients in each category was almost equal.

## Mode of delivery

Fifty-five patients had C/S: 18 emergencies and 37 elective C/S. In six of 37 patients who had elective C/S, the decision was based purely on the severity of their cardiac disease (Table 5).

**Table 5.** Indications For Caesarean Section

*Indication*	*Elective C/S (n 5 37)*	*Emergency C/S (n 5 18)*	*Severity of cardiac disease*	*No of patients*
Previous C/S	6	0		6
Twins	1	0		1
Breech	2	1 (came in in labour)		3
Failed induction of labour	0	4	mitral stenosis 1 ff balloon mitral valvulopasty / mitral stenosis 1.03 ff balloon mitral valvulopasty / mitral valve replacement 3 2 / congestive cardiac failure, ICU 0.8 / mixed mitral valve disease 3 3	8
Inadequate pelvis	6	0		6
Intra-uterine growth restriction	2	0	mitral valve replacement / mitral stenosis 1.6	2
Severe pre-eclampsia	1	1		2
Obstructive valval warts	3	0	mitral valve replacement 3 2 / mixed mitral valve disease	3
Cephelapelvic disproportion	0	1	mitral stenosis 1.2	1
Meconium-stained liquor	0	2	pre-eclampsia	2
Bad obstetric history	1	0	mixed mitral valve disease	1
Cardiomyopathy	1	0	ejection fraction 30	1
Critical/severe mitral stenosis	2	0	mitral stenosis 0.8/09	2
Severe aortic stenosis / mitral stenosis	1	0	0.9	1
Supraventricular tachycardia	1	0	aortic regurgitation / mitral regurgitation	1
Mitral stenosis / aortic stenosis	1	0		1
HIV infection	1	0		1

C/S = caesarean section.

## Impact of HIV infection

Thirty-one patients were HIV positive; 46 tested negative, and in the remaining 18, the HIV status was not known (declined testing after counselling). The demographic data and outcome measures were similar in HIV-infected and non-infected groups (Table 6). CD_4_ counts were < 200 cells/ml in three patients and all three patients and their babies showed no clinical evidence of infection. The mean CD_4_ count was 463 cells/ml (range 79−1 419).

**Table 6.** Impact Of HIV

*Parameter*	*HIV infected (n 5 31)*	*HIV uninfected (n 5 46)*
Age (years)	18–39	15–45
Parity		
P0	12	19
P1−3	18	25
Pre-term	13	14
NYHA class III−IV		
Intervention		
Balloon mitral valvuloplasty	4	4
Mitral valve replacement	1	
Haematoma	2	1
Mode of delivery		
Normal vaginal delivery	8	21
Caesarean section	21	25
Stillbirth	2	2
Birth weight (kg)		
< 2.5	9	13
> 2.5	22	33

NB: HIV status unknown in 18 patients.

## Discussion

Rheumatic heart disease (81%) was the most common heart disease complicating pregnancy in this audit. This finding is not unexpected, because it is well known that rheumatic fever is still a common problem in poorly resourced countries. Bhatla *et al.* and Abdel-Hady *et al.* reported similar findings in New Delhi, India and Egypt, respectively.^7^,^8^ This is in contrast to affluent societies in which congenital heart lesions are the dominant anatomical lesions in patients presenting with cardiac disease in pregnancy.^2^,^9^

Furthermore, differences in age at conception probably relate to earlier pregnancy with its attendant morbidity in poor countries. In this study, 54% of the patients were ≤ 25 years old, compared to a recent Canadian study where the mean age of patients was 32 years.^10^ Not only were the study patients younger at presentation for maternity care, but the initial detection of cardiac disease was made during pregnancy in 27 of the 95 patients, 17 of whom had valvular heart disease. Isolated mitral stenosis was present in 10 of these newly diagnosed ‘cardiacs’ (45.5%), similar to our previous report in which new cases of mitral stenosis accounted for 42% per year of the total number studied over a four-year period.^5^ This suggests that the incidence of mitral stenosis in our society has not changed; the condition is asymptomatic early in its natural history and is diagnosed when the majority of women are medically examined only during the first antenatal visit or when disease is of a severity that it produces symptoms.

There was a high rate of complications related to cardiac disease in this study (30%), the most common being congestive cardiac failure (14%), followed by arrhythmia (12%), and lastly, infective endocarditis (2%). Eight of the 13 patients with heart failure had valve stenoses and one had an obstructed mitral valve prosthesis. Mitral stenosis was the dominant heart lesion in five patients (23%) and prosthetic valve dysfunction was present in two. Non-valvular factors namely, anaemia (*n* = 4), urinary tract infection (*n* = 1) and severe pre-eclampsia (*n* = 1) were the precipitating factors for heart failure in these patients.

The high incidence of right heart failure associated with mitral stenosis in this study was not dissimilar to the 38% reported by Desai *et al.*, 31% by Silversides *et al.*, and others.^5^,^7^,^10^,^11^ Of importance, only one patient in this audit developed cardiac failure post-delivery. She was newly diagnosed with ventricular septal defect. Abdel-Hady *et al.*. reported that cardiac failure occurred post-delivery in 9.5% of patients.^8^ The lower incidence of cardiac failure post-delivery is probably due to the low admission threshold for cardiac disease, coupled with a high intervention rate for BMV in pregnancy.

Atrial fibrillation was present in nine patients (four with metallic valve prosthesis, four with severe mitral stenosis, and one with mixed mitral valve disease), in keeping with Malhotra *et al.* who reported a 7.3% incidence of arrhythmias.^12^ Our five patients with native valve lesions had left atrial sizes ranging from 50−77 cm and their pulmonary artery systolic pressures ranged from 37−71 mmHg. The risk of atrial fibrillation was obviously high in these patients, since they had metallic valves and the hypercoagulable state of pregnancy heightens the risk of valve dysfunction related to clot formation, which dictated the absolute need for adequate and precise anticoagulation. One patient in this series required mitral valve replacement during pregnancy for a thrombosed metallic prosthesis.

There was a high adverse event rate in patients with metallic valves. Half of them had complications, all related to the need for effective anticoagulation. However, unsupervised, effective anticoagulation comes with its attendant risk of warfarin embryopathy, spontaneous miscarriages, stillbirths and pre-term deliveries. 13-15 The incidence of embryopathy varies from 1.8−9.1%.^14^ Spontaneous miscarriages have been attributed to bleeding, loss of damaged foetuses and the patient’s pre-pregnancy disease.^16^ Embryopathy and the stillbirth rate have been found to be dose-dependent, with the incidence increasing in those who require warfarin above 5 mg daily.^17^

The perinatal mortality in our study was increased by the number of stillbirths (*n* = 5) that occurred in patients with rheumatic heart disease, especially those who were on oral anticoagulants, as well as the poor survival rate in foetuses with congenital malformations. The main factor was difficulty in achieving the target INR level leading to hypercoagulation. In addition, the presentation of these patients late in the second and third trimester did not permit change to heparin in the first trimester and led to the high stillbirth rate. Both foetal and maternal effects of anticoagulant therapy were frequent. Despite these problems, the perinatal mortality rate in this audit was 76/1 000 births, whereas the overall perinatal mortality rate in our unit is 90/1 000 births (department statistics). This was probably achieved by our careful surveillance policy in cardiac patients since we have transferred to the new unit.

The issue of which anticoagulant to use in pregnancy remains a controversy.^2^,^18^-^20^ In 1998, the American Heart Association/American College of Cardiology Task Force recommended warfarin in patients from one to 35 weeks’ gestation with older-generation mechanical prostheses. Newer low-profile prostheses are associated with lower risk and adjusted-dose subcutaneous heparin has been suggested.^21^ In a recent review, three regimens were recommended, namely, adjusted-dose low-molecular weight heparin throughout pregnancy; aggressive adjusted-dose unfractionated heparin throughout pregnancy; and unfractionated heparin or low-molecular weight heparin until the 13th week, with a change to warfarin until 36 weeks, when heparin is restarted.^19^ The last regimen is most often used at this centre because it is easy to monitor and cheaper than the other regimens.

There were no maternal deaths in this study, in stark contrast to the South African NCCEMD report where cardiac disease in pregnancy accounted for 43.3% of maternal deaths arising from pre-existing medical conditions.^4^ Other studies from resource-poor countries document a low but significant maternal mortality rate. Malhotra *et al.* reported two deaths in a series of 312 patients; Rahaman *et al.* one death in 229 patients, and Sawhey *et al.* 10 in 480 patients.^11^,^22^,^23^ Early hospitalisation, evaluation by a multidisciplinary approach, including cardiologists, together with closer monitoring probably contributed significantly to the fact that there were no maternal deaths in this study.

Only two patients required cardiac surgery in our study (clotted prosthesis and severe mitral regurgitation). In general, cardiac surgery is avoided during pregnancy because it carries a significant risk of foetal mortality and morbidity as a result of possible teratogenic effects of drugs during anaesthesia, disturbances in uteroplacental blood flow and embolic events to the uteroplacental circulation.^24^,^25^ In the past, surgery had a role in symptomatic cases of tight mitral stenosis, but this has now been superseded by BMV, which is effective and safer compared to open mitral valvulotomy and valve replacement.^10^,^25^,^26^ In our study, undetected mitral stenosis resulted in eight patients requiring intervention (BMV) during pregnancy. Recent studies show a decline in the rate of BMVs in pregnancy.^22^,^23^ Complications of BMV can be quite serious (mortality, cerebrovascular accident, cardiac perforation, mitral regurgitation and atrial septal defect) but fortunately, in experienced hands, these are low in frequency.^25^

The aim of the valvular heart disease classification into stenotic and regurgitant lesions was to define high-risk groups, and it is clear from this review that patients with metallic valves and those who had obstructive lesions did less well, despite advanced care. One obvious reason for this is that patients in all categories still continue to present with undetected valvular heart disease in the advanced stages of pregnancy. In total, 27 patients were diagnosed with heart disease for the first time in their history during pregnancy. Another point of note in the diagnosis is that the clinician infrequently mis-assesses the severity of disease. This is amply demonstrated by the fact that seven patients, assessed to have mixed mitral valve disease had, on Doppler-echocardiographic evaluation, tight mitral stenosis.

In this study, we searched for simple means of identifying ‘at risk’ cardiacs by practitioners, which would prompt earlier referral for echo-Doppler evaluation. Although there was a better correlation between the left atrial size and mitral valve area than PA pressure and mitral valve area, both correlations were weak and suggested that clinical evaluation to detect LA size, for instance by radiography, would not be as discriminative as one would expect in mitral valve disease. Add to this the difficulty of interpreting chest radiographs in the pregnant woman. This leaves the practitioner with the sobering reality that clinical assessment remains a prerequisite in identifying symptoms and signs suggestive of cardiac disease, which would determine the need for echocardiographic evaluation.

Another point of note is the seemingly high overall C/S rate in this audit (56%). In the majority (*n* = 47), the indications were purely obstetric, but in six patients it was based on the severity of the cardiac lesion. The high C/S rate is a reflection of the high-risk obstetric status of patients managed at this tertiary centre, since the overall C/S rate at IALCH is 70−80% (unpublished departmental data). Abdel-Hady *et al.* reported similar findings,^8^ whereas other authors^10^ have recorded lower C/S rates. The indications for C/S based on the severity of the disease were taken jointly by cardiologists and obstetricians, taking into account the echocardiographic findings and the clinical status of the patient at the time of decision making.

Lastly, we found that HIV-infected women did not have a higher wound infection rate or puerperal sepsis rate, nor was there a higher neonatal infection rate. There also did not appear to be any difference in outcome in the three women who had advanced disease with low CD_4_ counts (< 200 cells/ml). The standard of care during this study was the provision of single-dose nevirapine to the mother at the time of birth, and to the baby within 24 hours of delivery, for the prevention of mother-to-child transmission of the human immunodeficiency virus. The provision of antiretrovirals for maternal health had just begun at the time and the amounts available were too small. The incidence of HIV was high in this study but is in keeping with national sentinel antenatal surveillance studies in South Africa.

## Conclusion

This four-year audit of cardiac disease in pregnancy at a tertiary referral centre reveals a high rate of morbidity, with 23 patients (27%) having poor pregnancy outcomes. Monitoring of anticoagulant therapy in patients with metallic valve prosthesis following valve replacement needs to be improved to minimise foetal loss and maternal bleeding problems most often encountered in these patients. In addition, there was a significant misdiagnosis rate. Echo-Doppler evaluation by echocardiography accurately diagnosed the nature and severity of cardiac lesions, and so the initial diagnosis was changed in seven patients, emphasising the need for early referral for specialist care in cardiac cases as recommended by the Saving Mothers Report.^4^
